# Lipomatous Polyp Presenting With Intestinal Intussusception in Adults: Report of Four Cases

**DOI:** 10.4021/gr232e

**Published:** 2010-09-20

**Authors:** Shramana Mandal, Vibha Kawatra, Kajal Kiran Dhingra, Parul Gupta, Nita Khurana

**Affiliations:** a Department of Pathology, Maulana Azad Medical College, New Delhi-110002, India

**Keywords:** Lipomatous polyp, Intussusception, Intestine, Adults

## Abstract

Intussusception is a relatively common cause of intestinal obstruction in children but a rare, and uncommon clinical entity in adults accounting for 1%. Lipoma accounts for 4% of all benign tumors of the gut. Most of these are seen in the large intestine, usually submucosal and around ileocecal valve. These are often asymptomatic. Though these lesions are benign, it continues to present difficulties in the preoperative differentiation between malignant and benign colonic neoplasm.

## Introduction

Intussusception is a relatively common cause of intestinal obstruction in children but a rare, and uncommon clinical entity in adults accounting for 1%. Ninety-five percent (95%) of intussusception in children are idiopathic, whereas in adults only 7% is considered to be idiopathic, whenever presents prompt a clinical diagnosis of a malignant tumor [1, 2]. Surgical intervention is generally indicated as about half of both colonic and small intestinal intussusceptions are caused by malignant lesions [2]. Lipoma accounts for 4% of all benign tumors of the gut. Most of these are seen in the large intestine, usually submucosal and around ileocecal valve. These are often asymptomatic.

We present four cases of intestinal lipomatous polyp presenting with intussusception, of which three were involving the small intestine and one was present in the colon.

## Case Report

Four cases of intestinal lipomatous polyp were studied. The clinical, gross and microscopic findings were noted (Table 1). The age ranged from 45 to 60 years. The four cases presented with abdominal pain, recurrent vomiting and abdominal distention (features of intestinal obstruction).

**Table 1:** Clinical, Gross and Microscopic Findings of the Four Cases

	Age/Sex	Presentation	Location	Gross	Microscopy
Case 1	45/M	Pain, Abdominal distension, non passage of stools, flatus	Ileum, 14 cm from ileocolic junction	Ileoileal intussusseption; A polyp measuring 2 cm at the lading end of intussusceptions; 2 small proximal perforations	Submucosal Lipomatous polyp; Microscopic evidence of perforation
Case 2	55/M	Recurrent vomiting, abdominal distension	Ileum, 16 cm from ileocolic junction	Ileocolic Intussusceptions; Gangrene of the bowel loops; Small polyp 1 cm diameter	Gangrenous intestine; Lipomatous polyp of the small intestine; Microscopic evidence of perforation
Case 3	50/M	Vomiting abdominal distension, inability to pass stools for 10 days	24 cm from the Ileocolic junction, proximal perforation	Ileoileal intussusseption polyp 2.5 cm diameter	Gangrenous intestine; Lipomatous polyp of the small intestine; Mesentric artery thrombosis
Case 4	66/M	Sub acute intestinal obstruction	Ascending colon, 5 cm from Ileocolic junction	Colocolic intussusception; Polyp 3 cm diameter	Colonic Submucosal; Lipomatous polyp

**Figure 1:**
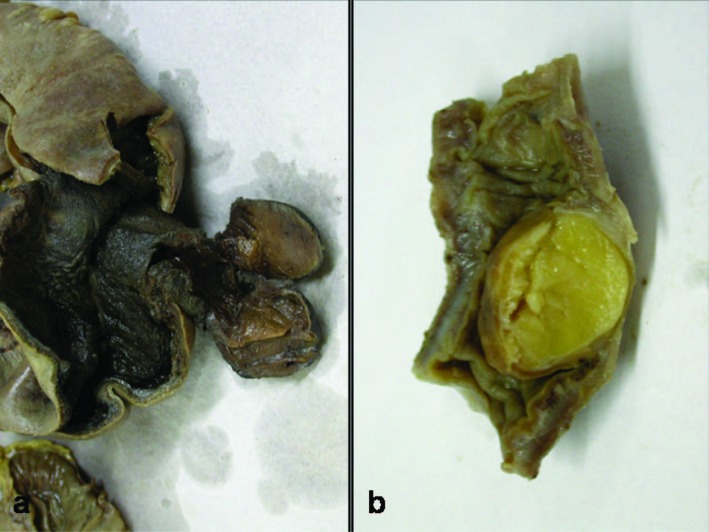
(a) Gangrene intestine with sesile Lipomatous polyp; (b) Submucosal Lipomatous polyp.

Grossly the polyp ranged in size from 1 to 3 cm. Ileoileal intussusception was identified in two cases (case 1 and 3), ileocolic intussusceptions in case 2 and colocolic intussusception in case 4. In all the cases the lesion was present in ileum except in one case (case 4) in which the polyp was present in the colon (Fig. 1). Perforation was identified in two cases (case 1 and 2) and gangrene in case 2 and 3.

**Figure 2:**
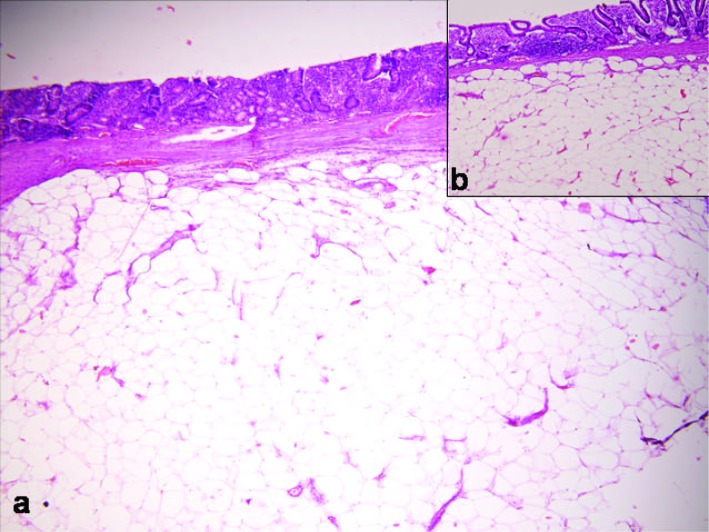
Thinned and stretcted out intestinal mucosa with submucosal lipomatous polyp (HE x 400).

Microscopically, the overlying mucosa of the intestine was thin and stretched out, and submucosal lipomatous polyp was identified in all four cases (Fig. 2). The polyp was well circumscribed and composed of mature adipocytes.

A final diagnosis of lipomatous polyp intestine with intussusception was made.

## Discussion

Intussusceptions in adults in 90% of the cases are caused by definable structural lesions, and about half of these lesions are malignant in both colonic and small intestinal intussusception. Lipomas are the commonest benign nonepithelial tumors of the colon and account for only 8-10% of all small intestinal intussusception in adults [3, 4].

Lipomas of the large bowel are reported as incidental findings in 0.3-0.5% of cases in autopsies. The commonest site for large bowel lipoma is the ascending colon, cecum, transverse colon (including both hepatic and splenic flexure), descending colon, sigmoid colon and the rectum [4]. The intestinal lipomas are generally submucosal and may protrude through the lumen, but they may also be subserosal in origin. These are generally found in fifth - sixth decade [4].

Adult intussusceptions present with nonspecific symptoms of bowel obstruction, including nausea, vomiting, and abdominal pain. Other symptoms may also be present such as melena, weight loss, fever, constipation, diarrhea, and abdominal mass [5]. Due to the vague symptoms and signs of adult intussusception, its preoperative diagnosis is difficult and surgical intervention is generally indicated as about half of both colonic and small intestinal intussusceptions which are caused by malignant lesions [2]. Colonic lipomas are generally asymptomatic, but occasionally patients may have intermittent crampy abdominal pain secondary to intussusception of a pedunculated lipoma or may present with intermittent fresh rectal bleeding. On barium enema lipomas appear as radiolucent mass (because of presence of fat), circular, ovoid, well demarcated, and smooth. Lipomas on barium enema show ‘squeeze sign’ due to their fluctuation in size and shape. The water enema, with water as the contrast agent, accentuates the difference in density between a lipoma and surrounding tissues. Lipomas of the large bowel can be seen, however, by colonoscopy. On computerized tomography (CT) scan the lipoma has a uniform appearance and density [4].

Pedunculated and sessile lesions can be removed endoscopically, but often large bowel lipomata are treated on the basis of a presumptive malignant diagnosis with exploratory laparotomy. Laparoscopic surgery is the treatment of choice for benign tumors of the small intestine because it is minimally invasive, with cosmetic, physical and economic benefits [6].

To conclude, colonic lipomas are rare, unusual, and continue to present difficulties in the preoperative differentiation between malignant and benign colonic neoplasm. Two cases of colonic lipomas are reported.

## References

[1] Triantopoulou C, Vassilaki A, Filippou D, Velonakis S, Dervenis C, Koulentianos E (2004). Adult ileocolic intussusception secondary to a submucosal cecal lipoma. Abdom Imaging.

[2] Lin MW, Chen KH, Lin HF, Chen HA, Wu JM, Huang SH (2007). Laparoscopy-assisted resection of ileoileal intussusception caused by intestinal lipoma. J Laparoendosc Adv Surg Tech A.

[3] Huang WS, Changchien CS, Lu SN (2000). Adult intussusception: a 12-year experience, with emphasis on etiology and analysis of risk factors. Chang Gung Med J.

[4] Marra B (1993). [Intestinal occlusion due to a colonic lipoma. Apropos 2 cases]. Minerva Chir.

[5] Azar T, Berger DL (1997). Adult intussusception. Ann Surg.

[6] Tsushimi T, Matsui N, Kurazumi H, Takemoto Y, Oka K, Seyama A, Morita T (2006). Laparoscopic resection of an ileal lipoma: Report of a case. Surg Today.

